# A Manual of Instruction in the Principles of Prompt Aid to the Injured

**Published:** 1889-05

**Authors:** 


**A Manual of Instruction in the Principles of Prompt Aid to the Injured.** Designed for military and civil use. By ALVAH H. DOTY, M. D., Major and Surgeon Ninth Regiment, N. G., S. N. Y.; Attending Physician to Bellevue Hospital Dispensary, New York. Cloth, 12mo, pp. xiii., 224. New York and London: D. Appleton & Co. 1889.

This work is designed for non-medical readers, and labors under the difficulty of explaining technical matters without the use of technical phrases. This always handicaps an author. We do not say this to condemn the attempt, but to call attention to the burden under which the author rests. The result must, of course, be considered in the light of what was intended. Judged in this way, we have no hesitation in saying, not that it supplies a long-felt want, but that in its matter it is as good as any manual of the kind we know, and in its illustrations and general arrangement superior to the most of them.

It may be divided into two parts: The first deals with what the author deems is sufficient for the laymen to know concerning the construction of the body and the functions of the various organs. He gives short descriptions of the bones, joints, cartilages, ligaments, membranes, the blood and the circulatory organs, respiration, alimentation and digestion, the kidneys, bladder, skin, spleen, and the nervous system. While, no doubt, the little knowledge given of these things may be of interest to those ignorant of them, we do not see that it serves a very good purpose detailed in this way. What is absolutely *necessary* to know could better be considered when treating of the various methods of giving prompt aid to the injured, as related in the rest of the book. Too little is given, or too much, according to the point of view. What benefit can the non-medical public receive from the cut on page 66, illustrating the sympathetic or ganglionic nervous system? This is taken as one of many examples suggestive of one of the faults of a work of this kind.

The latter part of the book describes, in a clear and concise manner, bandages and dressings, antiseptics, contusions and wounds, hemorrhage, fractures, dislocations and sprains, burns, scalds and frost-bites, unconsciousness, shock or collapse, brain injuries, apoplexy, etc., asphyxia and drowning, poisons and poisoning, convulsions of children, foreign bodies in the eye, ear, nose, larynx and pharynx, and transportation. In these chapters the author is at his best, and we feel sure that many will derive assistance from his pages.

The make-up of the book is excellent in every way—illustrations, printing, and paper being of the highest grade. Where a manual of this kind is required, no better one could be obtained.

